# 초등학교 고학년 남학생 어머니의 자녀 인유두종 바이러스 백신 접종의도 영향요인

**DOI:** 10.4069/kjwhn.2020.03.07

**Published:** 2020-03-18

**Authors:** Eun-Young Park, Tae-Im Kim

**Affiliations:** 1 Chungnam National University Hospital, Daejeon, Korea; 1 충남대학교병원; 2 Department of Nursing, Daejeon University, Daejeon, Korea; 2 대전대학교 간호학과

**Keywords:** Papillomaviridae, Papillomavirus vaccines, Mothers, Intention, 인유두종바이러스, 인유두종바이러스 백신, 어머니, 의도

## Abstract

**Purpose:**

The purpose of this study was to identify the factors influencing mothers’ intention to vaccinate their elementary-school sons against human papillomavirus (HPV).

**Methods:**

The participants were 151 mothers of fourth- to sixth-grade boys at three elementary schools in Daejeon, Korea. The data were analyzed using SPSS for Windows version 25.0. Descriptive statistics, the independent t-test, one-way analysis of variance, the Mann–Whitney U test, Pearson correlation coefficients, and multiple regression analysis were used.

**Results:**

The mothers’ score for intention to vaccinate their sons against HPV was 5.04 out of 7. Self-efficacy (β=.60, *p*<.001) and subjective norms towards HPV vaccination (β=.30, *p*<.001) were significant factors influencing mothers’ intention to vaccinate their elementary-school sons against HPV. These factors accounted for 81.0% of HPV vaccination intention among mothers (F=160.84, *p*<.001).

**Conclusion:**

The results of this study suggest that to increase the HPV vaccination rate for elementary-school boys, it is necessary to develop intervention strategies to improve mothers’ self-efficacy and subjective norms towards HPV vaccination and to verify the effects of those strategies. This research provides a foundation for designing interventions to increase the HPV vaccination rate of elementary-school boys.

## Introduction

### 연구 필요성

인유두종 바이러스(human papillomavirus, HPV)는 생식기 감염을 일으키는 가장 흔한 원인 중의 하나로, 현재까지 약 100여 종의 HPV가 발견되었으며 이 중 40여 종은 직접적인 성적 접촉을 통해 감염된다. HPV 감염은 대부분 별다른 증상 없이 자연적으로 소실되지만, 고위험군 HPV에 반복적으로 노출되는 경우 여성의 자궁경부암, 외음부암, 질암뿐만 아니라 남성의 생식기 사마귀, 직장암, 음경암 및 두경부암 등을 일으키는 주요 원인으로 보고되고 있다[1]. 실제 HPV가 각종 암 발생에 기여하는 정도는 자궁경부암은 거의 100%이고, 항문암 88%, 질암 78%, 음경암 50%, 구강인두암 30.8%, 외음부암 24.9%이며, 세계적으로 HPV 감염과 관련하여 연간 약 63만 건에 달하는 암이 남녀에게 발생하고 있는 것으로 보고되고 있다[2]. 그러나 이 질환들은 대부분 HPV 백신 접종을 통해 예방이 가능하기 때문에 남녀 모두에서 HPV 백신 접종의 중요성이 강조되고 있다[3].

HPV 백신은 HPV 감염 예방을 위한 가장 효과적이고 안전한 방법으로 알려져 있다[1,4,5]. 특히 HPV는 성적 접촉을 통해 전파되므로 HPV 감염으로 인해 유발되는 암 발생과 이로 인한 사회경제적 부담을 감소시키고 남녀의 성 건강을 유지, 증진하기 위해서는 남녀 모두가 HPV 백신을 접종해야 한다[6]. 그럼에도 불구하고 우리나라에 HPV 백신이 처음 소개될 때 자궁경부암 예방 백신으로 소개되어 주로 여성에게 권고되고 있으며[3], 남성에게는 불필요한 것으로 인식하여 남성의 HPV 백신 접종률이 여성에 비해 매우 저조하다[7,8]. 국내 남아의 HPV 백신 접종률은 1.6% [9]로 미국의 남아 접종률 56.5% [10]에 비해 매우 낮다. 더욱이 우리나라를 포함한 대부분의 국가에서 HPV 백신 무료 접종을 여아를 대상으로 실시하고 있어 남아의 HPV 접종률을 높이기 위한 보다 적극적인 대책 마련이 필요하다.

HPV 감염은 성관계 시작과 더불어 증가하므로 이를 예방하기 위해 HPV 백신 접종 시기는 첫 성경험이 이루어지기 이전이 가장 이상적이다. 또한 HPV 백신은 성인에서 3회 접종하는 반면 12세 이하에서는 2회 접종만으로도 높은 면역효과를 나타내기 때문에 보건학적, 경제적 측면에서 12세 이하에 HPV 백신 접종을 시행하는 것이 비용 효과적이다[1,6]. 최근 우리나라 청소년의 첫 성관계 연령은 2011년 13.8세에서 2018년 13.6세로 낮아진 반면 성관계 경험률은 2011년 4.9%, 2018년 5.7%, 2019년 5.9%로 점차 증가하고 있음을 고려할 때[11], 이들의 HPV 감염 예방을 위해 HPV 백신 접종 최적 연령인 11–12세에 HPV 백신을 접종할 수 있도록 권고하는 것은 시급히 시행해야 할 당면 과제이다. 특히 자녀의 연령이 어린 경우 어머니는 자녀의 HPV 백신 접종 수행에 결정적 역할을 하는 것으로 알려져 있다[12-14]. 따라서 자녀(아들)의 백신 통제권을 가지고 있는 어머니의 HPV 백신 접종의도 및 관련 요인을 연구하는 것은 자녀(아들)의 HPV 백신 접종률 향상을 위한 전략 마련에 기초자료가 될 것이다.

한편, 계획된 행위 이론(theory of planned behavior, TPB)에 의하면 특정 행동에 대한 행동의도는 행위의 직접적 결정인자로 행위에 선행하며, 행동의도는 3가지 핵심요인인 행위에 대한 태도(attitude), 주관적 규범(subjective norm) 및 지각된 행위 통제(perceived behavior control)에 의해 결정된다. 즉, 특정 건강행동이 가치 있는 결과를 낳는다고 생각할수록, 그 행동을 수행할 능력과 기회가 있다고 생각할수록 그 행동을 실제로 할 가능성이 높아짐을 의미한다[15]. 국내외 선행연구에서 HPV 백신에 대한 태도, 주관적 규범, 지각된 행위 통제를 부모의 자녀 HPV 백신 접종의도를 예측하는 주요 변수로 보고하며[13-14,16] TPB가 HPV 백신 접종의도를 설명하는 이론으로 널리 활용되고 있다. 그러나 이들 세 가지 변수만으로는 행동의도를 충분히 설명하는 데 한계가 있어 다른 추가변수를 포함해야 한다는 주장이 있는데, 지각된 행위 통제는 자기 효능감과 유사한 개념으로 제시되었고[15], 지식[12,16-18]과 자기 효능감[14,19]은 HPV 백신 접종의도를 예측하는 주요 변수로 보고되고 있다.

어머니 혹은 부모의 HPV 백신 접종의도와 관련된 국외 선행연구들을 살펴보면, 남아 부모의 자녀 HPV 백신 접종의도 및 영향요인을 확인하는 연구가 최근 활발하게 진행되고 있다[8,12,18,20]. 반면에 국내 선행연구는 12–14세 여자 청소년 어머니를 대상으로 한 연구 보고[21], HPV 백신 접종 최적 연령인 9–12세 여아 어머니를 대상으로 한 연구 보고[19,22] 등이 있다. 그러나 초등학교 고학년 남학생 어머니를 대상으로 한 연구는 경기도와 강원도 소재 초등학교 5–6학년 남학생의 학부모를 대상으로 한 Kang [16]의 연구 보고 이외에는 찾아보기 힘들어 남아 어머니의 HPV 백신 접종의도에 대한 경험적 근거가 충분치 않은 실정이다.

이에 본 연구는 HPV 백신 접종 최적 연령인 대전 소재 3개 초등학교에 재학 중인 4–6학년 남학생 어머니의 자녀(아들) HPV 백신 접종의도 및 관련 요인을 규명함으로써 초등학교 고학년 남학생 어머니의 자녀(아들) HPV 백신 접종의도를 높이고, 궁극적으로 남학생의 HPV 백신 접종률을 높이기 위한 중재 프로그램 개발 시 기초자료로 활용하고자 한다.

### 연구 목적

본 연구의 목적은 초등학교 고학년 남학생 어머니의 HPV 지식, 자녀(아들) HPV 백신 접종에 대한 태도, 주관적 규범, 자기 효능감 및 HPV 백신 접종의도를 파악하고, HPV 백신 접종의도에 미치는 영향요인을 확인하기 위함이며 구체적인 연구목적은 다음과 같다.

1) 대상자의 일반적 특성 및 HPV 관련 특성을 파악한다.

2) 초등학교 고학년 남학생 어머니의 HPV 지식, 자녀(아들) HPV 백신 접종에 대한 태도, 주관적 규범, 자기 효능감 및 자녀 HPV 백신 접종의도를 파악한다.

3) 초등학교 고학년 남학생 어머니의 자녀(아들) HPV 백신 접종의도와 HPV 지식, 자녀 HPV 백신 접종에 대한 태도, 주관적 규범, 자기 효능감과의 상관관계를 파악한다.

4) 초등학교 고학년 남학생 어머니의 자녀(아들) HPV 백신 접종의도에 영향을 미치는 요인을 파악한다.

## Methods

Ethics statement: This study was approved by the Institutional Review Board of Daejeon University (1040647-201812-HR-008). Informed consent was obtained from the subjects.

### 연구 설계

본 연구는 초등학교 고학년 남학생 어머니의 HPV 지식, 자녀(아들) HPV 백신 접종에 대한 태도, 주관적 규범, 자기 효능감이 자녀(아들) HPV 백신 접종의도에 미치는 영향을 확인하기 위한 서술적 조사연구이다.

### 연구 대상

본 연구의 대상자는 대전 소재 초등학교 3개교를 임의 선정하여 4–6학년에 재학 중인 남학생(363명)의 어머니 전수를 대상으로 하였다. 본 연구에 필요한 대상자 수는 G*Power 3.2.1 프로그램을 사용하여 효과 크기 0.15 (중간 크기), 유의수준 .05, 검정력 90%, 일반적 특성 및 HPV 백신 관련 변수를 포함하여 회귀분석에 필요한 독립변수 10개를 투입한 결과 최소 표본 수는 147명으로 산출되었다. 배포된 363부 중 175부가 회수되었으며(회수율 48.2%), 이들 중 응답이 불충분한 24부를 제외한 151부를 최종 분석에 사용하였다.

### 연구 도구

#### HPV 지식

HPV 지식은 Kim과 Ahn [23]이 대학생을 대상으로 개발한 HPV 지식 측정도구를 메일을 통해 도구 개발자로부터 도구 사용에 대한 승인을 받은 후 사용하였다. 이 도구는 총 20문항으로 구성되었으며, 고위험 HPV와 저위험 HPV 감염 결과, 관련 질환, 잠복기, 예후, 면역력과의 관련성, 전염경로, 검사와 진단, 예방과 치료 및 선천성 감염 등의 내용을 포함하고 있다. 각 문항에 대해 ‘그렇다’, ‘아니다’, ‘모르겠다’로 평가하며, 점수의 범위는 0–20점이다. 정답은 1점, 오답과 ‘모르겠다’에 응답한 경우 0점 처리하였고, 점수가 높을수록 HPV 지식수준이 높음을 의미한다. 도구 개발 당시 신뢰계수 Cronbach’s α는 .87이었고[23], 본 연구에서 HPV 지식도구의 신뢰계수 Kuder-Richardson Formula 20 (KR-20)의 r값은 .74였다.

#### HPV 백신 접종에 대한 태도

HPV 백신 접종에 대한 태도는 Gerend와 Shepherd [14]가 개발한 TPB 구성개념 측정도구(Assess TPB Constructs) 중 HPV 백신 접종에 대한 태도 측정도구를 Kim [24]이 번역하여 수정, 보완한 도구를 사용하여 측정하였다. 도구는 메일을 통해 도구 개발자의 사용 승인을 받은 후 사용하였다. 이 도구는 대상자의 자녀(아들) HPV 백신 접종을 통해 얻을 수 있는 이익에 대한 대상자의 긍정적 또는 부정적 평가를 측정하는 4문항으로 구성되어 있다. HPV 백신 접종에 대한 태도는 Likert 7점 척도로 ‘매우 동의한다’ 7점에서 ‘전혀 동의하지 않는다’ 1점을 배점하여 합산한 점수를 의미하며, 점수가 높을수록 HPV 백신 접종에 대한 태도가 긍정적임을 의미한다. Gerend와 Shepherd [14]의 도구 개발 당시 신뢰계수 Chronbach’s α는 .88이었고, Kim [24]의 연구에서는 .93이었으며, 본 연구에서는 .92였다.

#### HPV 백신 접종에 대한 주관적 규범

HPV 백신 접종에 대한 주관적 규범은 Gerend와 Shepherd [14]가 개발한 TPB 구성개념 측정도구 중 HPV 백신 접종에 대한 주관적 규범 측정도구를 Kim [24]이 번역하여 수정, 보완한 도구를 사용하여 측정하였다. 도구는 메일을 통해 개발자의 사용 승인을 받은 후 사용하였다. 이 도구는 대상자의 부모, 의사, 친구들이 HPV 백신 접종을 권고하는 것에 대한 대상자의 규범적 신념의 정도를 측정하는 6문항으로 구성되어 있다. Likert 7점 척도로 각 문항에 대해 ‘매우 동의한다’ 7점에서 ‘전혀 동의하지 않는다’ 1점을 배점하여 합산한 점수를 의미하며, 점수가 높을수록 HPV 백신 접종에 대한 주관적 규범이 높음을 의미한다. Gerend와 Shepherd [14]의 도구 개발 당시 신뢰계수 Chronbach’s α는 .78이었고, Kim [24]의 연구에서는 .92, 본 연구에서는 .90이었다.

#### HPV 백신 접종에 대한 자기 효능감

HPV 백신 접종에 대한 자기 효능감은 Gerend와 Shepherd [14]가 개발한 TPB 구성개념 측정도구 중 HPV 백신 접종에 대한 자기 효능감 측정도구를 Kim [24]이 번역하여 수정, 보완한 도구를 사용하여 측정하였다. 도구는 메일을 통해 도구 개발자의 사용 승인을 받은 후 사용하였다. 이 도구는 주변의 어려움에도 불구하고 자녀(아들)에게 HPV 백신 접종을 하고자 하는 대상자의 자기 확신 혹은 자신감의 정도를 측정하는 총 6문항으로 구성되어 있으며, Likert 7점 척도로 각 문항에 대해 ‘매우 동의한다’ 7점에서 ‘전혀 동의하지 않는다’ 1점을 배점하여 합산한 점수로, 점수가 높을수록 자녀(아들) HPV 백신 예방접종에 대한 자기 효능감이 높음을 의미한다. Gerend와 Shepherd [14]의 도구 개발 당시 신뢰계수 Chronbach’s α는 .85이었고, Kim [24]의 연구에서 .97이었으며, 본 연구에서는 .85였다.

#### HPV 백신 접종의도

HPV 백신 접종의도는 Gerend와 Shepherd [14]가 개발한 TPB 구성개념 측정도구 중 HPV 백신 접종의도 측정도구를 Kim [24]이 번역하여 수정, 보완한 도구를 사용하여 측정하였다. 도구는 메일을 통해 개발자로부터 사용 승인을 받은 후 사용하였다. 이 도구는 어머니가 자녀에게 HPV 백신을 접종하기 위해 정보를 얻고 노력하며, 의료인의 권유 시 자녀(아들)에게 HPV 백신을 접종할 가능성에 관한 5문항으로 구성되어 있다. Likert 7점 척도로 각 문항에 대해 ‘매우 동의한다’ 7점에서 ‘전혀 동의하지 않는다’ 1점을 배점하여 합산하였으며, 점수가 높을수록 자녀(아들) HPV 백신 접종의도가 높음을 의미한다. Gerend와 Shepherd [14]의 도구 개발 당시 신뢰계수 Chronbach’s α는 .96이었으며, Kim [24]의 연구에서 .96, 본 연구에서 .86이었다.

### 자료 수집

자료수집은 연구자가 사전에 해당 초등학교를 방문하여 학교장과 보건교사에게 연구목적과 방법 등을 설명하고 연구수행에 대한 동의를 구하였다. 그 후 보건교사는 학생들에게 연구목적과 내용이 담긴 설명문, 연구 참여 동의서, 설문지 및 회신용 밀봉지(양면 테이프가 부착된 대봉투)를 봉투에 넣어 밀봉된 상태로 배부하여 어머니들에게 전달하였다. 연구 참여 동의서에 자발적으로 서명한 어머니들이 동봉한 설문지를 작성하고 연구 참여 동의서와 함께 밀봉 처리하여 보건교사에게 제출하는 방식으로 자료를 회수하였으며, 연구에 참여한 모든 어머니들에게는 감사의 표시로 소정의 선물을 제공하였다. 자료수집 기간은 2019년 3월 1일부터 2019년 4월 10일까지였다.

### 자료 분석

수집된 자료는 IBM SPSS Statistics for Windows ver. 25.0 (IBM Co., Armonk, NY, USA) 프로그램을 이용하여 대상자와 자녀의 일반적 특성, HPV 감염 및 HPV 백신 관련 특성을 기술 통계를 이용하여 분석하였다. 대상자의 HPV 지식, 자녀(아들) HPV 백신 접종에 대한 태도, 주관적 규범, 자기 효능감 및 접종의도도 기술 통계를 이용하여 분석하였으며, 대상자의 HPV 지식, 자녀(아들) HPV 백신 접종에 대한 태도, 주관적 규범, 자기 효능감 및 접종의도 간의 상관관계는 Pearson’s correlation coefficients으로, HPV 백신 관련 특성에 따른 자녀(아들) HPV 백신 예방접종 의도의 차이는 독립표본 t-test와 one way ANOVA, Mann–Whitney U test를 이용하여 분석하였다. 대상자의 자녀(아들) HPV 백신 접종의도에 영향을 미치는 요인을 확인하기 위해 다중회귀분석(multiple regression analysis)을 실시하였다.

## Results

### 대상자의 일반적 특성, HPV 감염 및 HPV 백신 관련 특성

대상자의 평균 연령은 42.74±3.19세였으며, 41–45세가 90명(59.6%)으로 가장 많은 분포를 나타내었고, 학력은 대졸 이상이 139명(92.1%)이었으며, 직업이 있는 대상자가 104명(68.9%)이었다. 종교가 있는 대상자는 72명(47.7%)이었고, 평균 월 수입은 500만 원 이상이 88명(58.3%)으로 가장 많았다. 자녀는 5학년이 88명(58.3%)으로 가장 많았고, 어머니가 생각하는 자녀의 HPV 백신 접종 최적 연령은 평균 14.85±2.45세였으며, 백신 접종 최적 연령인 13세 이하라고 응답한 수는 49명(32.5%)이었다.

대상자의 자궁경부암 가족력은 ‘없다’가 145명(96.0%)이며, 부인과 질환 경험은 ‘없다’가 121명(80.1%)이었다. Papanicolaou (Pap) test 검사 경험은 ‘있다’가 123명(81.5%)이었고, 이 중 100명(81.3%)은 규칙적으로 Pap test 검사를 하고 있는 것으로 나타났다. 조사 대상 어머니 중 HPV 감염에 대해 들어본 경험이 있는 어머니는 111명(73.5%)이었고, HPV 백신에 대해 들어본 경험이 있는 어머니는 89명(58.9%)이었다. 자녀(아들) HPV 백신 접종 여부는 비접종이 151명(100%)이었다. 어머니의 HPV 백신 접종 여부는 접종이 15명(9.9%)이었고, 자녀(아들) HPV 백신 접종계획은 103명(68.2%)에서 자녀에게 HPV 백신을 접종할 계획을 가지고 있었다. 이 중 6개월 이내에 백신 접종계획을 갖고 있는 대상자는 19명(18.4%)이었다. 자녀(아들) HPV 백신 접종계획이 없다고 응답한 48명의 대상자 중 HPV 백신 접종을 하지 않으려는 이유는 ‘잘 몰라서’가 23명(47.8%)으로 가장 많았고, 다음으로 ‘백신의 부작용에 대한 우려’ 12명(25.0%), ‘백신의 효과에 대한 불신’ 9명(18.8%)의 순으로 나타났다. 의료보험 적용 시 HPV 백신 접종계획은 ‘예’가 115명(76.2%)이었다(Table 1).

### 대상자의 HPV 지식, 자녀(아들) HPV 백신 접종에 대한 태도, 주관적 규범, 자기 효능감 및 HPV 백신 접종의도

대상자의 HPV 지식은 총 20점 만점에 평균 11.13±2.19점이었다. 대상자의 자녀 HPV 백신 접종에 대한 태도는 7점 만점에 평균 평점 5.25±1.15점, 주관적 규범은 7점 만점에 평균 평점 4.47±1.56점, 자기 효능감은 7점 만점에 평균 평점 4.94±1.44점, HPV 백신 접종의도는 7점 만점에 평균 평점 5.04±1.26점이었다(Table 2).

### 대상자의 HPV 지식, 자녀(아들) HPV 백신 접종에 대한 태도, 주관적 규범, 자기 효능감 및 HPV 백신 접종의도의 상관관계

대상자의 자녀(아들) HPV 백신 접종의도는 HPV 지식(r=.26, *p*=.001), 자녀(아들) HPV 백신 접종에 대한 태도(r=.65, *p*<.001), 주관적 규범(r=.79, *p*<.001), 자기 효능감(r=.88, *p*<.001)과 통계적으로 유의한 양의 상관관계가 있는 것으로 나타났다(Table 3).

### 대상자의 일반적 특성, HPV 감염 및 HPV 백신 관련 특성에 따른 자녀(아들) HPV 백신 접종의도

대상자의 일반적 특성, HPV 감염 및 HPV 백신 관련 특성에 따른 자녀(아들) HPV 백신 접종의도의 차이를 검정한 결과, 통계적으로 유의한 차이를 나타낸 변수는 없는 것으로 나타났다(Table 4).

### 대상자의 자녀(아들) HPV 백신 접종의도 영향요인

대상자의 자녀(아들) HPV 백신 접종의도에 영향을 미치는 요인을 확인하기 위하여 HPV 백신 접종의도와의 상관관계 검정에서 연구 대상 어머니의 자녀(아들) HPV 백신 접종의도와 유의한 상관관계를 나타낸 HPV 지식, 자녀 HPV 백신 접종에 대한 태도, 주관적 규범, 자기 효능감을 독립변수로 투입시켜 다중회귀분석을 실시하였다. 다중회귀분석을 실시하기 전 본 연구 회귀모형의 가정을 검정한 결과 회귀분석을 위한 모든 가정이 충족되었다. 즉, 잔차의 산포도를 분석한 결과 잔차의 분포는 ‘0’을 중심으로 균등하게 흩어져 있어 회귀식의 선형성과 등분산성의 가정을 충족하였다. 히스토그램과 회귀 표준화 잔차의 정규 P-P 도표를 검정한 결과도 잔차가 45도 직선에 근접함으로써 오차의 정규분포와 등분산성의 가정을 만족하였다. 오차의 자기상관을 나타내는 Durbin–Watson 통계량은 1.83으로 비교적 2에 가까워 오차항의 독립성 및 자기상관에 문제가 없었다. 독립변수 간의 상관관계는 .41에서 .88로 나타났고, 공차한계(tolerance)는 모든 독립변인에 대해 .29에서 .95로 모두 .1 이상이었으며, 분산팽창인자(variance inflation factor)도 1.06–3.51로 모두 10을 넘지 않아 독립변수 간 다중공선성 문제는 없는 것을 확인하였다. 분석 결과 어머니의 자녀 HPV 백신 접종의도에 영향을 미치는 변인은 자녀(아들) HPV 백신 접종에 대한 자기 효능감(β=.60, *p*<.001), 주관적 규범(β=.30, *p*<.001)이었고, 이들 요인들로 대상자의 자녀(아들) HPV 백신 접종의도를 81.0% 설명하는 것으로 나타났으며, 회귀방정식의 모형도 유의한 것으로 확인되었다(F=160.84, *p*<.001) (Table 5).

## Discussion

본 연구는 TPB를 적용하여 초등학교 고학년 남학생 어머니의 자녀(아들) HPV 백신 접종의도 및 이에 대한 영향요인을 파악하고자 시도되었다.

연구 결과 초등학교 고학년 남학생 어머니의 자녀 HPV 백신 접종의도는 HPV 지식, HPV 백신 접종에 대한 태도, 자기 효능감, 주관적 규범과 유의한 양적 상관관계가 있었으며, 이들 중 자기 효능감, 주관적 규범은 초등학교 고학년 남학생 어머니의 자녀 HPV 백신 접종의도에 유의한 영향을 미치는 변수로 확인되었다. 본 연구에서 나타난 주요 결과를 중심으로 논의하고자 한다.

본 연구에서 초등학교 고학년 남학생의 HPV 백신 접종률은 0%였다. 국내 선행연구에서 HPV 백신 접종률은 12–14세 남아에서 3.1% [16], 13–17세 남아에서 1.6% [9], 14세 미만 여아에서 42.1–59.4% [19,21,25]로 남학생의 HPV 백신 접종률이 여학생에 비해 현저히 낮다. 국외 남학생의 HPV 백신 접종률 역시 여학생에 비해 낮았으나[7,8], 미국의 13–17세 남아의 HPV 백신 접종률 56.5% [10]와 비교하면 국내 남학생의 백신 접종률이 현저히 낮은 것을 알 수 있다. HPV 감염이 남녀 모두에게 흔히 발생되는 성 전파성 질환으로 이를 예방하려면 남녀 모두 HPV 백신을 접종해야 비용 효율적임을 고려해 볼 때[3,6,9], 남학생의 HPV 백신 접종률 향상을 위한 노력이 절대적으로 필요하다. 특히 국내 남학생의 HPV 백신 접종률이 낮은 이유는 HPV 감염 및 HPV 백신에 대한 홍보 부족 및 관련 교육 프로그램이 부재하고[9], 남학생에게는 HPV 백신 국가 무료 예방접종이 아직 제도적으로 정착되지 않았으며[9,16], HPV 백신이 처음 소개될 때 자궁경부암 예방 백신으로 소개되어 여성에게 국한되는 접종이라고 생각하는 남학생 어머니의 인식이 반영되어 나타난 결과[9,16]라 생각해 볼 수 있다. 남아의 HPV 백신 접종에 의료보험 혜택이 주어지는 경우 본 연구 대상 어머니의 자녀 HPV 백신 접종계획이 68.2%에서 76.2%로 증가하였고, HPV 백신을 국가 필수 예방접종 항목으로 지정한 결과 HPV 백신 접종률이 향상되었다는 국외 연구보고[26]는 국가에서 제도적으로 남학생을 HPV 백신 무료 예방접종 대상자로 포함한다면 남학생의 HPV 접종률을 어느 정도 향상시킬 수 있음을 시사한다.

본 연구 대상 어머니들이 생각하는 자녀의 HPV 백신 접종 최적연령은 평균 14.9세였고, 자녀 HPV 백신 접종계획이 있는 어머니 중 ‘6개월 이내에 백신을 접종하겠다’에 응답한 어머니는 18.4%에 불과하였다. 선행연구에서 어머니들이 생각하는 자녀 HPV 백신 접종 최적 시기로 초등학교 여학생 어머니는 13세 이상이 60.0% [22], 여중생 어머니는 17.7세[27], 12–14세 여학생 부모는 14세 이상이 80.4% [21]로, 14세 미만 연령대는 자녀 HPV 백신 접종 시기로 아직 이르다고 생각하고 있음을 알 수 있다[12,13]. 이는 11–12세가 HPV 백신 접종 최적기로 권고되는[1,4] 현 시점에서 어머니들의 인식 개선을 위한 적극적인 노력이 요구되고 있음을 시사한다. 앞서 말했듯 HPV 백신 접종은 성 생활을 시작하기 전에 실시하는 것이 가장 이상적이고[1], 12세 이하에서는 2회 접종만으로도 높은 면역력을 나타내므로 이 시기에 접종하는 것이 비용 효율적이기 때문이다[4,6]. 한국 청소년의 첫 성 경험 평균 연령은 13.6세이고[11], 자녀의 연령이 어린 경우 어머니는 자녀의 HPV 백신 접종에 결정적 역할을 하는 것으로 알려져 있다[12-14]. 따라서 초등학교 남학생의 HPV 백신 적기 접종률을 증가시키기 위한 어머니 대상 교육 프로그램 개발 시에 HPV 백신 접종 시기, 안정성 및 긍정적 효과에 대한 내용을 포함하여 어머니들의 인식 전환을 유도할 필요가 있다[12,19].

HPV 백신 접종의도는 백신 접종행위를 유도하는 강력한 예측요인으로 알려져 있다[8,12-14]. 본 연구 결과 어머니의 자녀 HPV 백신 접종의도는 7점 만점에 5.04점(100점 만점에 72.0점)으로 중간 수준보다 다소 높은 것으로 나타났다. 비록 대상 아동의 연령대가 다르고 동일한 도구로 측정을 하지 않아 직접 비교하는 데 제한이 있으나, 국내외 선행연구에서 17세 미만 남아 어머니의 자녀 HPV 백신 접종의도가 100점 만점에 66.2–76.6점[8,9,16]으로 보고된 결과와 유사한 수준이다. 그러나 실제 남아의 HPV 백신 접종률은 본 연구 결과를 포함하여 0–3.1% [9,16]로 매우 저조한 것을 고려해 볼 때 이에 대한 원인을 규명하고 어머니의 자녀 HPV 백신 접종의도를 실제 자녀의 HPV 백신 접종으로 연결시키기 위한 전략이 필요하다.

본 연구 결과 연구 대상 어머니의 자녀(아들) HPV 백신 접종의도는 HPV 지식, 자녀(아들) HPV 백신 접종에 대한 태도, 주관적 규범, 자기 효능감과 통계적으로 유의한 양의 상관관계가 있는 것으로 나타났다. 그러나 이들 변수를 투입하여 대상자의 자녀(아들) HPV 백신 접종의도에 영향을 미치는 요인을 확인한 결과, 자녀(아들) HPV 백신 접종에 대한 자기 효능감과 주관적 규범이 어머니의 자녀(아들) HPV 백신 접종의도에 영향을 미치는 요인으로 확인되었고, 이들 변수로 대상자의 자녀(아들) HPV 백신 접종의도를 81.0% 설명하는 것으로 나타났다. 이와 같은 결과는 비록 TPB를 설명하는 모든 변수가 유의한 영향을 미치지는 않았으나, 자기 효능감과 주관적 규범이 행동의도를 예측하는 변인임을 검증함으로써 확장된 TPB의 기본 가정에 대한 경험적 근거를 축적한 점에서 의의가 있다.

HPV 백신 접종에 대한 자기 효능감은 HPV 백신 접종 시 장애를 극복할 수 있는 개인의 능력에 대한 판단을 의미한다. 자기 효능감은 TPB에서 지각된 행위 통제와 높은 상관관계를 나타내어 이를 반영하는 지표로 널리 활용되고 있으며, 예방접종과 같은 예방적 건강행위를 선택하고 이를 지속하는 데 영향을 미치는 예측인자로 보고되고 있다[14,28]. 본 연구 대상 어머니의 HPV 백신 접종에 대한 자기 효능감은 7점 만점에 평균 평점 4.94점(100점 만점에 70.6점)으로, 연구 대상 어머니들은 자녀(아들) HPV 백신 접종에 대해 비교적 용이하고 자신감 있게 지각하고 있는 것으로 나타났다. 측정도구가 달라 직접 비교는 어렵지만 동일 연령대 초등학생 어머니의 자녀(아들) HPV 백신 접종에 대한 자기 효능감이 100점 만점에 65.8점[16], 만 12세 여아 어머니의 자녀(딸) HPV 백신 접종에 대한 자기 효능감이 100점 만점에 66.4점[19]이라고 보고한 선행연구와 유사한 수준이다. 또한 본 연구에서 자기 효능감은 어머니의 자녀(아들) HPV 백신 접종의도를 예측하는 가장 영향력 있는 요인으로 확인되어, 자기 효능감이 개인의 HPV 백신 접종의도 및 접종행동을 잘 예측한다는 선행연구 보고를 지지하였다[8,13,14,16,19]. 선행연구에서 어머니들의 HPV 백신 접종에 대한 자기 효능감이 높을수록 자녀 HPV 백신 접종의도가 증가하였고[14,16,19], 백신 접종과 같은 예방행위는 백신 관련 지식 수준이 높고 교육을 많이 받은 사람일수록 많이 하는 것으로 보고되고 있다[17,19]. 본 연구 대상 어머니의 HPV 지식은 100점 만점에 55.7점으로 저조하였고, HPV 백신을 접종하지 않는 이유 중 ‘HPV 백신에 대한 정보 부족과 부작용에 대한 우려’가 72.8%였다. 이는 국내 선행연구[16,19,21,22]에서도 일관되게 보고되고 있다. 실제 HPV 백신의 부작용은 일반 예방접종으로 인한 부작용과 크게 다르지 않으며 안전성이 보고되고 있음을 고려해 볼 때[3] 어머니들은 HPV 감염이나 백신의 효과에 대해 정확한 지식을 갖고 있지 않음을 알 수 있다. 비록 본 연구에서 HPV 지식이 어머니의 자녀 HPV 백신 접종의도를 예측하는 변인에 포함되지 않았으나 자기 효능감이 행동으로 연결되기 위해서는 개인이 과제를 수행하는 데 필요한 지식과 기술을 소유하고 있어야 한다[29]. 따라서 남학생의 HPV 백신 접종률을 높이려면 어머니의 HPV 백신 접종에 대한 자기 효능감 증진을 위한 중재 전략 마련과 더불어, HPV 감염 및 HPV 백신과 관련된 정확한 정보를 제공하여야 할 것이다.

본 연구 결과 대상자의 자녀(아들) HPV 백신 접종에 대한 주관적 규범은 7점 만점에 4.47점(100점 만점에 63.9점)으로 Kang [16]의 연구에서 보고한 주관적 규범 65.8점과 유사한 수준이었다. 즉, 연구 대상 어머니들이 자녀(아들)의 HPV 백신 접종에 대해 주변 사람들로부터 받는 사회적 압력을 중간 수준 이상으로 지각하고 있음을 알 수 있다. 또한 백신 접종의도 영향요인 확인을 위한 회귀분석 결과 주관적 규범은 자기 효능감 다음으로 초등학교 고학년 남학생 어머니의 자녀(아들) HPV 백신 접종의도를 예측하는 요인으로 나타났다. 이는 주관적 규범이 백신 접종의도를 결정하는 주요 예측요인이라는 선행연구 보고[8,13,14,16,27]를 지지하는 결과이다. 주관적 규범은 예방접종과 같은 예방적 건강행위를 선택하거나 결정할 때 영향을 미치는 요인으로 알려져 있다[12,14]. 특히 예방적 건강행위 선택과 관련된 의사결정 초기 단계에서 관련 정보나 활용 가능한 프로그램이 부족하고 개인이 자신의 결정에 대한 확신이 적은 경우, 개인에게 영향력이 있는 주변 사람들이나 학교 보건교사, 의료인의 권고가 예방적 건강행위 선택에 결정적인 영향을 미치는 것으로 보고되고 있다[10,13,30]. 실제 의료인에게 HPV 백신 접종에 대한 권고를 받은 경우 부모의 자녀 HPV 백신 접종의도 및 백신 접종률이 증가한 보고[7,30]가 이를 뒷받침해 주고 있다. 따라서 초등학교 고학년 남학생 어머니의 HPV 백신 접종에 대한 주관적 규범을 높이기 위한 전략으로 의료인의 권고와 보건교사의 학부모 참관 수업, 가정통신문을 통한 홍보와 교육 및 대중매체를 활용한 교육과 홍보 등을 고려해 볼 수 있다[13,16,19]. 또한 국가적 차원에서 HPV 백신 무료 접종 제도를 남학생에게도 확대 실시하여 남성도 HPV 관련 질병으로부터 보호받고 나아가 남녀의 성 건강과 삶의 질 향상에도 기여할 수 있도록 구체적이고 적극적인 홍보 전략을 마련해야 할 것이다.

한편, 연구 대상 어머니의 HPV 지식과 HPV 백신 접종에 대한 태도는 자녀(아들) HPV 백신 접종의도와 유의한 상관관계가 있었으나 HPV 백신 접종의도 예측요인에는 포함되지 않았다. 비록 측정도구가 동일하지 않아 직접 비교는 어렵지만, TPB 기반으로 TPB 변인에 HPV 지식을 포함하여 어머니의 자녀 HPV 백신 접종의도 예측요인을 확인한 국내 연구[16,21]에서 어머니의 HPV 지식은 어머니의 자녀 HPV 백신 접종의도 예측요인에서 제외되었으며, 태도, 주관적 규범, 지각된 행위 통제는 국내외 선행연구[8,13,16,21,27]에서 어머니의 자녀 HPV 백신 접종의도 예측요인으로 제시된 바 있다. 어머니의 HPV 지식이 자녀 HPV 백신 접종의도 예측요인에서 제외된 것은 본 연구 및 선행연구[16,21]에서 어머니의 HPV 지식 점수가 44.5–56.3점으로 매우 낮았고, 남아의 HPV 백신 접종은 최근에 이르러서야 주목되고 있어 충분히 홍보가 되어 있지 않으며, 남아 혹은 그들 어머니를 대상으로 한 공식적인 교육 프로그램이 없는 등의 이유로 남아 HPV 백신 접종에 대한 인식이 충분치 않아 어머니의 자녀(아들) HPV 백신 접종의도로 연계되지 않았을 가능성을 고려해 볼 수 있다[9,12]. 어머니의 HPV 백신 접종에 대한 태도는 일부 선행연구[13,16,27]에서 어머니의 HPV 백신 접종의도를 예측하는 강력한 변인으로 제시되었으며, 국외 일부 선행연구[8,14]에서는 영향력이 낮은 것으로 보고되었다. 이는 자료수집이 진행된 지역과 대상자의 일반적 특성 및 HPV 관련 특성의 차이가 어느 정도 영향을 미쳤을 것이라 생각되며, 후속 연구를 통해 이를 규명해야 할 것이다.

본 연구는 대전 소재 3개 초등학교 남학생 부모를 대상으로 편의 표집하였고, 설문지 회수율이 낮아 자료수집 대상 집단의 대표성을 충분히 확보하지 못했으므로 본 연구 결과를 확대 해석하는 데 신중을 기해야 한다. 연구의 목적이 어머니의 자녀(아들) HPV 백신 접종의도 및 관련 요인을 파악하는 것이기 때문에 실제 자녀(아들) HPV 백신 접종행위를 예측하는 데 한계가 있다. 또한 자료수집 시 자기보고식 설문지로 이루어져, 자기보고식 설문지가 갖는 대상자의 응답 편향을 배제할 수 없다는 제한점이 있다.

그러나 본 연구는 국내 경험적 근거가 충분치 않은 초등학교 고학년 남학생 부모를 대상으로 HPV 지식, 자녀(아들) HPV 백신 접종에 대한 태도, 주관적 규범, 자기 효능감 및 HPV 백신 접종의도 간의 관계를 파악하고, HPV 백신 접종의도에 영향을 미치는 요인을 규명함으로써 HPV 백신 접종의도를 증진시키기 위한 효율적인 중재 전략을 수립하는 데 필요한 기초자료를 제공한 점에서 간호 실무에 기여하였다. 또한 자녀(아들) HPV 백신 접종에 대한 자기 효능감과 주관적 규범이 자녀(아들) HPV 백신 접종의도에 영향을 미치는 요인임을 규명함으로써 확장된 TPB의 기본가정에 대한 경험적 근거를 축적한 점에서 간호이론과 연구에 기여하였다.

## Conclusion

본 연구는 TPB에 근거하여 초등학교 고학년 남학생 어머니의 HPV 백신 접종의도, HPV 지식, HPV 백신 접종에 대한 태도, 자기 효능감, 주관적 규범 간의 관계를 파악하고, HPV 백신 접종의도에 미치는 영향요인을 확인하기 위해 시도하였다. 본 연구에서 대상자의 HPV 예방접종 의도에 유의한 영향을 미치는 요인은 자녀 HPV 백신 접종에 대한 자기 효능감과 주관적 규범으로 확인되었다. 따라서 HPV 백신 접종의 적기인 초등학교 고학년 남학생 어머니의 자녀 HPV 백신 접종의도를 효과적으로 증진시키고 나아가 남아의 HPV 백신 접종률을 높이기 위해서는 HPV 백신 접종에 대한 자기 효능감, 주관적 규범을 강화할 수 있는 중재 전략을 마련할 필요가 있다. 즉, 자녀(남아) HPV 백신 접종에 대한 어머니들의 자기 효능감을 증진하기 위해 HPV 백신 접종에 대한 정확한 정보를 제공하고 적극적인 홍보를 통해 백신 접종의 장애요인을 줄이며, 주관적 규범 증진을 위해 의료인과 보건교사에 의한 교육과 권고를 실시해야 할 것이다. 또한 국가적 차원에서 초등학교 고학년 남아에게도 HPV 백신 접종비용을 보조하거나, 같은 연령대의 여학생의 경우처럼 무료로 접종하는 등 국가 차원의 정책적 제도 마련이 필요하다.

본 연구 결과를 토대로 다양한 지역의 초등학교에 재학 중인 남학생의 어머니를 대상으로 반복 연구를 실시하여 어머니들의 HPV 백신 접종의도 영향요인에 대한 경험적 근거를 축적할 것을 제언한다. 또한 본 연구에서 영향요인으로 확인된 HPV 백신 접종에 대한 자기 효능감과 주관적 규범을 강화하는 중재 프로그램을 개발하고 그 효과를 검증하는 후속 연구를 제언하는 바이다.

## Figures and Tables

**Table 1.** General, HPV infection-, and HPV vaccination-related characteristics of participants (N=151)

Variable	Categories	n (%)
*General characteristics*		
Age (year)	≤ 40	33 (21.9)
	41–45	90 (59.6)
	46-50	28 (18.5)
	Mean ± SD 2.74 ± 3.19	
Education	≤ High school	12 (7.9)
	University	109 (72.2)
	≥ Graduate	30 (19.9)
Job	Yes	104 (68.9)
	No	47 (31.1)
Religion	Yes	72 (47.7)
	No	79 (52.3)
Monthly income (× 10,000, KRW)	< 300	21 (13.9)
	300–500	42 (27.8)
	≥ 500	88 (58.3)
Son’s school year	4th grade	18 (11.9)
	5th grade	88 (58.3)
	6th grade	45 (29.8)
*HPV infection-, and HPV vaccination-related characteristics*		
Appropriate age to vaccinate son against HPV (year)	≤ 13	49 (32.5)
	≥ 14	64 (42.4)
	No response	38 (25.1)
Family history of cervical cancer	Yes	6 (4.0)
	No	145 (96.0)
Experience with gynecological diseases	Yes	30 (19.9)
	No	121 (80.1)
Experience with Pap test	Yes	123 (81.5)
	No	28 (18.5)
Receive Pap test regularly (n = 123)^†^	Yes	100 (81.3)
	No	23 (18.7)
Awareness of HPV infection	Yes	111 (73.5)
	No	40 (26.5)
Awareness of HPV vaccine	Yes	89 (58.9)
	No	62 (41.1)
Son’s HPV vaccination status	Yes	0 (0.0)
	No	151 (100)
Mother’s HPV vaccination status	Yes	15 (9.9)
	No	136 (90.1)
HPV vaccination plan for son	No	48 (31.8)
	Yes	103 (68.2)
	Within 6 months	19 (18.4)
	After 6 months	84 (81.6)
Reasons not to vaccinate son against HPV (n = 48)^‡^		
	Lack of information about HPV vaccination	23 (47.8)
	Concern about the side effects of the vaccine	12 (25.0)
	Distrust of the effectiveness of the vaccine	9 (18.8)
	High cost	2 (4.2)
	It is not necessary for a boy	2 (4.2)
Plan to vaccinate son against HPV under health insurance coverage	Yes	115 (76.2)
	No	36 (23.8)

HPV: Human papillomavirus; KRW: Korean won; Pap: papanicolaou.

† Mothers who had received a Pap test,

‡ Excludes mothers who planned to vaccinate sons.

**Table 2.** HPV-related knowledge, attitudes, subjective norms, self-efficacy, and intention to vaccinate son against HPV vaccination among participants (N=151)

Variable	Mean ± SD	Number of items	Item range	Possible range
HPV knowledge	11.13 ± 2.19	20	0–1	1–20
Attitude toward son’s HPV vaccination	5.25 ± 1.15	4	0–7	0–28
Subjective norms related to son’s HPV vaccination	4.47 ± 1.56	3	0–7	0–21
Self-efficacy related to son’s HPV vaccination	4.94 ± 1.44	3	0–7	0–21
Intention to vaccinate son against HPV	5.04 ± 1.26	5	0–7	0–35

HPV: Human papillomavirus.

**Table 3.** Relationships among HPV-related knowledge, attitudes, subjective norms, self-efficacy, and intention to vaccinate son against HPV among participants (N=151)

Variable	r (*p*)			
	Attitude toward son’s HPV vaccination	Subjective norms related to son’s HPV vaccination	Self-efficacy related to son’s HPV vaccination	Intention to vaccinate son against HPV
HPV knowledge	.20 (.013)	.02 (.012)	.02 (.007)	.26 (.001)
Attitude toward son’s HPV vaccination	1	.53 (< .001)	.70 (< .001)	.65 (< .001)
Subjective norms related to son’s HPV vaccination		1	.77 (< .001)	.79 (< .001)
Self-efficacy related to son’s HPV vaccination			1	.88 (< .001)

HPV: Human papillomavirus.

**Table 4.** Mothers’ intention to vaccinate sons against HPV by general, HPV infection- , and HPV vaccination-related characteristics (N=151)

Variable	Categories	Mothers’ intention to vaccinate sons against HPV		
		Mean ± SD	t or F or Z	*p*
*General characteristics*				
Age (year)	≤ 40	27.12 ± 4.78	1.98	.141
	41–45	24.60 ± 6.81		
	46–50	24.93 ± 6.01		
Education level	≤ High school	28.25 ± 6.27	1.67	.192
	University	24.80 ± 6.25		
	≥ Graduate	25.50 ± 6.43		
Job	Yes	25.29 ± 6.31	0.22	.826
	No	25.04 ± 6.39		
Religion	Yes	25.34 ± 6.59	0.29	.768
	No	25.03 ± 5.97		
Monthly income (× 10,000, KRW)	< 300	26.05 ± 6.85	0.37	.827
	300–500	25.76 ± 4.92		
	≥ 500	24.73 ± 6.97		
Son’s school year	4th grade	25.72 ± 4.95	2.80	.064
	5th grade	24.24 ± 6.35		
	6th grade	26.91 ± 6.47		
*HPV infection- and HPV vaccination-related characteristics*				
Appropriate age to vaccinate son against HPV (year)	≤ 13	27.18 ± 5.34	1.77	.079
	≥ 14	25.26 ± 5.31		
Family history of cervical cancer	Yes	27.83 ± 7.93	–1.31^†^	.189
	No	25.10 ± 6.25		
Experience with gynecological diseases	Yes	24.17 ± 6.73	–1.14^†^	.253
	No	25.47 ± 6.21		
Experience with Pap test	Yes	25.55 ± 6.20	–1.25^†^	.210
	No	23.71 ± 6.71		
Receive Pap test regularly (n = 123)	Yes	26.00 ± 6.12	–1.49^†^	.134
	No	23.61 ± 6.30		
Awareness of HPV infection	Yes	25.42 ± 6.48	0.68	.495
	No	24.63 ± 5.88		
Awareness of HPV vaccine	Yes	25.26 ± 6.75	0.11	.914
	No	25.15 ± 5.68		
Mother’s HPV vaccination status	Yes	27.93 ± 5.07	–1.56^†^	.119
	No	24.91 ± 6.38		

HPV: Human papillomavirus; KRW: Korean won; Pap: papanicolaou.

† Mann-Whitney U test.

**Table 5.** Factors influencing mothers’ intention to vaccinate sons against HPV (N=151)

Variable	B	SE	β	t (*p*)
(Constant)	.75	.26		2.88 (.005)
Self-efficacy	.52	.06	.60	8.93 (< .001)
Subjective norms	.41	.05	.30	5.35 (< .001)
Attitudes	.06	.06	.06	1.16 (.249)
HPV knowledge	.03	.02	.06	1.63 (.106)
	Adjusted R²=.81, F (*p*)=160.84 (<.001)			

HPV: Human papillomavirus.
